# Fecal microbiota transplantation in a patient with chronic diarrhea and primary and secondary immunodeficiency (common variable immunodeficiency and splenectomy)

**DOI:** 10.3389/fcimb.2024.1456672

**Published:** 2024-09-30

**Authors:** Katarzyna Napiórkowska-Baran, Jarosław Biliński, Małgorzata Pujanek, Paweł Hałakuc, Antoni Pietryga, Bartłomiej Szymczak, Aleksander Deptuła, Tomasz Rosada, Zbigniew Bartuzi

**Affiliations:** ^1^ Department of Allergology, Clinical Immunology and Internal Diseases, Collegium Medicum Bydgoszcz, Nicolaus Copernicus University, Torun, Poland; ^2^ Human Biome Institute, Gdańsk, Poland; ^3^ Department of Gastroenterology, Collegium Medicum Bydgoszcz, Nicolaus Copernicus University, Torun, Poland; ^4^ Student Research Club of Clinical Immunology, Department of Allergology, Clinical Immunology and Internal Diseases, Collegium Medicum Bydgoszcz, Nicolaus Copernicus University, Torun, Poland; ^5^ Department of Propaedeutics of Medicine and Infection Prevention, Ludwik Rydygier Collegium Medicum in Bydgoszcz, Nicolaus Copernicus University in Torun, Bydgoszcz, Poland

**Keywords:** fecal microbiota transplantation, gut microbiota, inborn errors of immunity (IEI), primary immunodeficiencies (PID), common variable immune deficiency (CVID), secondary immunodeficiency (SID), splenectomy

## Abstract

The gut microbiota serves a crucial role in the development of host immunity. Immunocompromised patients are particularly vulnerable to dysbiosis not only by virtue of a defect in the immune system but also due to increased susceptibility to infection and multiple courses of antibiotic therapy. Fecal microbiota transplantation is by far the most effective option for restoring gastrointestinal homeostasis. However, it is contraindicated in patients with significant primary and secondary immunodeficiencies. This article presents the case of a 59-year-old patient with common variable immunodeficiency, after splenectomy at age 39 for primary immune thrombocytopenia, who manifested diarrhea of up to 10 stools per day accompanied by secondary malnutrition and cachexia. The patient was admitted to the hospital on multiple occasions due to this condition, with stool PCR tests confirming a HHV-5 (*Cytomegalovirus*, CMV) infection. Following the administration of valganciclovir, the patient’s complaints diminished, although, upon cessation of the drug, the symptoms recurred. In addition, the patient had an intestinal infection with *C. difficile* etiology. Given that the patient’s therapeutic options had been exhausted, after obtaining informed consent from the patient and approval from the bioethics committee to conduct a medical experiment, treatment of diarrhea was undertaken by fecal microbiota transplantation with the certified preparation Mbiotix HBI from the Human Biome Institute. The patient underwent two transplants, with a one-week interval between them. The initial procedure was performed using the endoscopic method, while the subsequent was conducted using the capsule method. Following the administration of the applied treatment, the patient’s symptoms were successfully alleviated, and no adverse effects were observed. A microbiological analysis of the intestinal microbiota was conducted prior to and following transplantation via next-generation sequencing (NGS). No recurrence of symptoms was observed during the two-year follow-up period. To the best of our knowledge, this is the first fecal microbiota transplantation in an adult patient with primary and secondary immunodeficiency.

## Introduction

Patients with primary immunodeficiencies, currently known as inborn errors of immunity (IEI), exhibit an elevated propensity towards autoimmunity and neoplasia, but most predominantly infections, including those affecting the gastrointestinal tract (Notarangelo et al., 2020). The etiological agents of infection may vary depending on the specific type of deficiency. The disruption of gastrointestinal tract homeostasis in patients with IEI is not solely attributable to the immunodeficiency itself but is also influenced by the pharmacological agents employed, including immunosuppressants and antimicrobial drugs (Castagnoli et al., 2021). Due to an increased susceptibility to infection, patients are subjected to a multitude of antibiotic therapies, including broad-spectrum antibiotics. Probiotics are contraindicated in certain patients, including those with impaired cellular immunity, those with a central vascular catheter in place, and those taking selected immunosuppressive drugs. Unfortunately, due to the lack of randomized trials, probiotic manufacturers often do not recommend their use in all patients with primary and secondary immunodeficiencies, regardless of the defect type.

Immunodeficiency disorders involving aberrant antibody production represent the most prevalent IEIs, accounting for 50-60% of all primary deficiencies (Shin et al., 2020). Among these, common variable immunodeficiency is the most clinically significant. It is observed with a frequency of 1:25,000 to 1:50,000 in the global human population. Patients diagnosed with this deficiency require lifelong immunoglobulin replacement therapy. These patients present with deficiencies in IgA, IgG, and, optionally, IgM, as well as reduced levels of memory B lymphocytes and other deficiencies. Unfortunately, there is currently no causal treatment for the deficiency. Consequently, the optimal approach is to address the complications as effectively as possible (Bonilla and Geha, 2009).

## Case report

The patient, aged 59, was referred to the Immunology Outpatient Clinic at the age of 55 following a suspected diagnosis of inborn error of immunity. Based on the medical history, physical examination and serological test results, in accordance with the criteria of the European Society for Immunodeficiencies (ESID), a diagnosis of common variable immunodeficiency was established. The patient was qualified for life-long immunoglobulin replacement therapy (IgRT). Furthermore, the patient presented with a plethora of chronic conditions associated with the deficiency, including autoimmune hemolytic anemia, primary immune thrombocytopenia (for which the patient underwent splenectomy at age 39), liver cirrhosis and esophageal varices resulting from primary cholangitis, pernicious anemia, and malnutrition. Moreover, the patient was treated for hypertension and paroxysmal supraventricular tachycardia. In addition to splenectomy, the patient underwent appendectomy at the age of 35 and electroresection of the prostate due to hypertrophy at the age of 53. The patient received chronic subcutaneous human immunoglobulin substitution using a hyaluronidase-assisted regimen at a dose of 30 g/4 weeks, which equates to 0.45 g/kg body weight/4 weeks, as part of the Drug Program for the treatment of IEI at the Hematology Department in his area of residence. The patient’s other medications include carvedilol 3.25 mg 2x1 tab, ursodeoxycholic acid 250 mg taken 2x/day (2-0-1), mesalazine 1000 mg 2x1 tab, methylprednisolone 4 mg 1x1 tab, tamsulosin hydrochloride 0.4 mg 1x1 tab, vitamin B12 1x100 µg/4 weeks i.m. and torsemide 5 mg 1x1 tab.

The patient had been experiencing chronic diarrhea since he was 54 years old, with approximately ten stools per day. The implementation of replacement immunoglobulin therapy did not result in any improvement in the number of stools passed. Consequently, the patient had been admitted to the hospital multiple times in his local area, where PCR stool tests confirmed cytomegalovirus (CMV) infection. The symptoms abated after valganciclovir treatment but subsequently recurred following the discontinuation of the drug (the patient was prescribed the maintenance dose of 450 mg twice a day for a period of six months after the last exacerbation). Furthermore, the patient had previously experienced a *C. difficile* infection and had a history of gastrointestinal colonization with the NDM-type carbapenemase-producing *K. pneumoniae*. Parenteral nutrition was administered during the patient’s hospitalization due to diarrhea. At the follow-up appointment at the Immunology Clinic, in light of the patient’s persistent diarrhea and secondary malnutrition, a consultation at the Nutrition Clinic was recommended, and the implementation of permanent parenteral nutrition was advised. Ultimately, the patient was deemed eligible for this form of nutrition, which resulted in increased body weight and improved nutritional exponents, yet the diarrhea persisted. Endoscopic examination of the intestine and histopathological examination of the biopsy specimens excluded the diagnosis of inflammatory bowel disease (IBD).

At the age of 58, the approval of the Bioethics Committee at Collegium Medicum in Bydgoszcz (KB 579/2021) was obtained, and an attempt was made to treat the patient’s diarrhea by fecal microbiota transplantation (FMT) with the certified preparation Mbiotix HBI from the Human Biome Institute. The patient underwent two FMT procedures with an interval of one week. The first procedure used the endoscopic method, while the second involved the capsule method (7 Jun 2022 and 14 Jun 2022). The Mbiotix HBI was prepared from stool samples coming from two different donors, D4 for the first and D7 for the second FMT. Prior to the procedure, a gastrointestinal decontamination protocol was implemented, entailing the oral administration of the following antibiotics for five days: gentamicin 3x80 mg, colistin 4x2 million IU, and vancomycin 4x125 mg. On the sixth day, the bowels were cleansed in a manner similar to that employed for colonoscopy, with the administration of macrogol and sodium sulfate (4 sachets of 75 grams each dissolved in 4 liters of water). On the seventh day (i.e., the day of the initial FMT), the entire Mbiotix preparation was administered into the ileocecal region. Following the procedure, the patient was turned from side to side for approximately 30 minutes to facilitate the retention of the material. Subsequently, seven days later, following the cleansing of the bowel using the aforementioned method, another FMT was performed; however, this time, the capsule method was utilized, i.e., the oral route.

**Figure 1:**
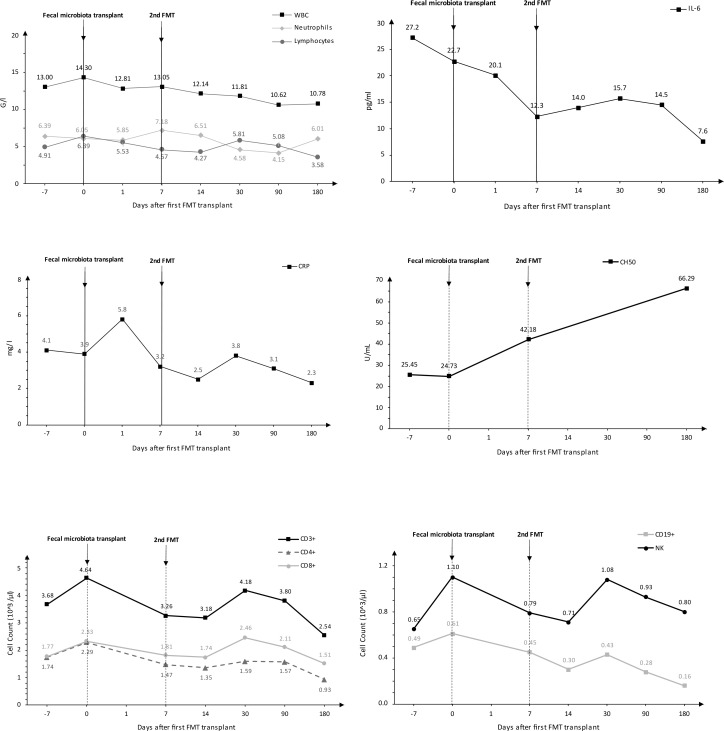
Analysis of selected biochemical and immunological tests conducted on the patient.

**Figure 2:**
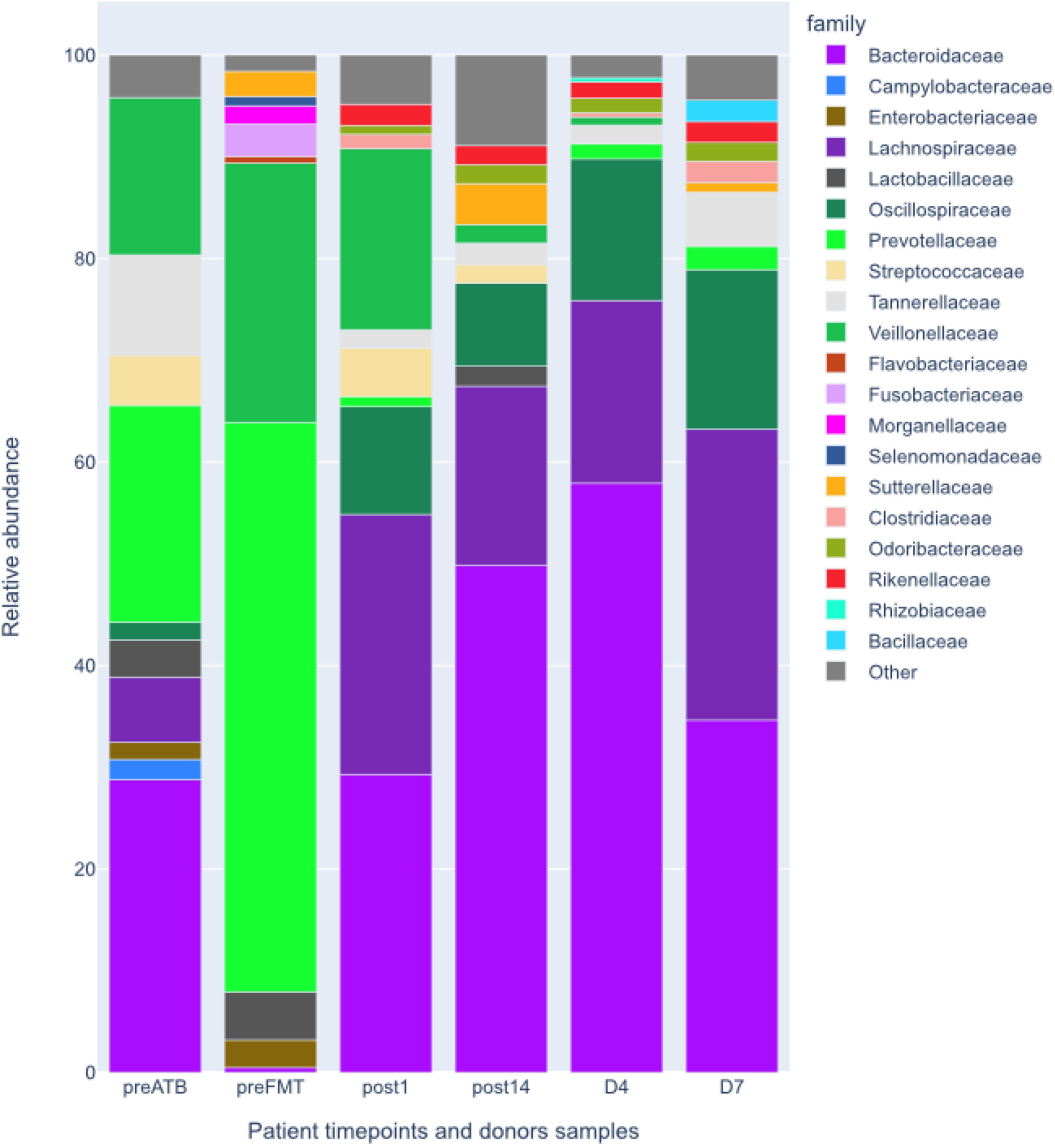
Relative abundances at family level of patient’s intestinal microbiota before and after the FMT and donors’ intestinal microbiota shown for comparison. preATB -before antibiotic therapy, preFTM - before Fecal Microbiota Transplantation; post1 - one day after FMT; post14 - fourteen days after FMT; D7 – donor stool used for first FMT; D4 – donor stool used for second FMT.

**Figure 3:**
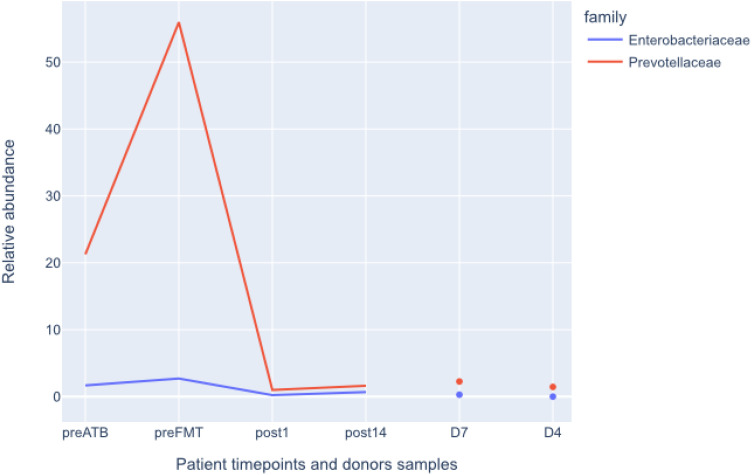
Relative abundances of *Enterobacterales* and *Pevotellaceae* before and after FMT. Donors shown for comparison. preATB -before antibiotic therapy, preFTM - before Fecal Microbiota Transplantation; post1 - one day after FMT; post14 - fourteen days after FMT; D7 – donor stool used for first FMT; D4 – donor stool used for second FMT.

Following the FMT procedure, the patient’s clinical condition improved, with stools occurring at a frequency of 1-2 times per day. Furthermore, the patient reported the cessation of continuous cold sensation symptoms. During the one-year follow-up period, there was no increase in the frequency of stools. The results of the tests performed on the patient before and after FMT are presented in **Figure 1**. The results of the microbiological analysis of stools performed by the NGS method are shown in **Figures 2**, **3**. During the two-year follow-up period, no recurrence of the symptoms was observed.

## Materials and methods

The patient underwent laboratory examinations and stool microbiological tests prior to and following the fecal microbiota transplantation. The patient gave written consent to participate in the study. The study protocol was approved by the Ethical Committee of the Collegium Medicum in Bydgoszcz (KB 579/2021).

### Laboratory analysis

During each hospitalization and follow-up visits to the clinic, blood samples were collected from the vein in the cubital fossa in order to determine valuable parameters through following tests: complete blood count with smear, C-reactive protein (CRP), interleukin 6 (IL-6), hemolytic activity of complement (CH50), lymphocyte phenotyping (CD19+, CD3+, CD4+, CD8+, CD56+). Blood was collected from the median cubital vein using a closed vacuum system (Vacuette, Greiner Bio-One).

### Stool analysis- collection, storage, isolation, sequencing

Stool samples were collected from the patient at following timepoints: before antibiotic treatment (preATB), before the FMT (preFMT), and 24 hours and 14 days after the procedure (post1 and post14). In all cases samples were placed in a sterile container and frozen to -20 degrees Celsius. The samples were transported to on dry ice and stored at -80 degrees Celsius, until handing out to external company (genXone, Poland) for DNA isolation and sequencing. Subsamples of donor stool samples used for preparation of Mbiotix HBI were handled in the same way. Feces were thawed on ice and the DNA was isolated using Genomic Mini AX Stool Biotome 4nPore kit (A&A Biotechnology). Sequencing libraries were prepared using Ligation Sequencing gDNA kit v14 (Oxford Nanopore Technologies) following manufacturers protocol with following minor modifications. Firstly, 700ng of DNA per sample was used instead of 400ng. Furthermore, at the DNA repair stage incubation times were extended from 5min at 20 degree C and 65 degree C to 20 mins at both temperatures. Acquired libraries were sequenced on PrometheION using R10.4.1 flowcells. In majority of cases more than one flowcell was used with the goal of total sequencing depth of 20 Gbp per sample.

At all metagenomic samples were processed independently. Generated raw reads were basecalled by guppe basecaller ver. 6.4.2 using super-accurate mode. Quality of basecalled reads have been evaluated using NanoPlot ver 1.41.0. Host reads have been removed by mapping to human reference genome (GRCh38_noalt_as) by minimap2 ver 2.24. Surviving metagenomic reads were assembled firstly by MetaFlye ver 2.9.1. The initial assembly was followed by four rounds of polishing by minimap2 and Racon ver 1.4.20 to correct potential assembly errors.

Abundance of acquired contigs was estimated based on reads mapped to final assemblies by minimap2. Kraken2 was used to assign a taxonomic label to each contig using Kraken 2 RefSeq database. Relative abundances of taxa were estimated as percentage of total length or reads mapped to contigs classified as particular taxon. Additionally all reads have been mapped by minimap2 against HHV-5 (human *Cytomegalovirus)* reference genome (GCA_000845245.1) to verify its presence.

Open reading frames have been predicted using Prodigal ver 2.6.3. All acquired protein sequences were analyzed using AMRFinderPlus to identify antibiotic resistance genes (ARG) and genes responsible for resistance to other environmental factors. Genes abundances were reported as RPKMs to avoid biases from contig and gene lengths.

## Results

### Laboratory tests

Fecal microbiota transplantation caused a decrease in IL-6 concentration and an increase in CH50 values. Due to the research conducted on one patient and the significantly limited number of measurements before and after transplantation, statistical analysis was not performed. If it were made, it would be of questionable quality and the possible results would be highly erroneous. The results of the laboratory tests performed are presented in **Figure 1**.

### Microbiological/bioinformatics analysis

Acquired total sequencing depth is comparable for all patients and donors with >90% surviving host removal (**Table 1**). Acquired metagenome assemblies are different in size with assemblies acquired preATB and preFMT samples being two smallest (155,5 and 75,5Mbp respectively). Similarly, the general taxonomic composition of preATB and preFMT samples can be distinguished from the general taxonomic composition of post FMT (post1 and post14) and donor samples (D4 and D7). In the latter, the majority of microbiome consists of members of *Bacteroidaceae* (e.g. *Bacteroides* and *Phocaeicola*), *Lachnospiraceae* (e.g. *Roseburia* and *Blautia*) and *Oscillospiraceae* (*Faecalibacterium*), typical for health gut microbiome (**Figure 2**). However, *Prevotella* and *Streptococcus* form a major part of the microbiome in the preATB sample and especially the preFMT sample (**Figures 2**, **3**).

**Table 1:** Overall overview of metagenomic data quality and quantity.

	preATB	preFMT	post1	post14	D7	D4
# reads	4544146	5017694	3960081	6169520	2973709	4053569
Reads total length	20,4 Gbp	19,2 Gbp	24,2 Gbp	27,0 Gbp	29,3 Gbp	27,9 Gbp
Reads mean length	4492,6	3959,1	6255,8	4458,7	9876,4	6888,3
% non-host reads	98,8%	90,1%	98,1%	98,4%	99,7%	99,7%
Assembly total length	155,5 mln	75,5 mln	344,6 Mbp	408,1 Mbp	360,9 Mbp	344,9 Mbp

preATB -before antibiotic therapy, preFTM - before Fecal Microbiota Transplantation; post1 - one day after FMT; post14 - fourteen days after FMT; D7 – donor stool used for first FMT; D4 – donor stool used for second FMT.

Due to the patient’s medical history all samples were specifically checked for presence of *Klebsiella pneumoniae*. In all cases relative abundances were close to detection level (<0,1%). The only confidently detected member of *Enterobacterales* was *Escherichia coli* present in the preFMT sample (2,1%). Similar check was performed for other known human gut pathogens, e.g. *Clostridioides difficile*, *Giardia intestinalis*, *Cryptosporidia*, with none being present at >0,1% of relative abundance. Not a single contig in any sample was classified as HHV-5 (*Cytomegalovirus*). Since HHV-5 infection was previously confirmed by PCR, all reads were mapped to the HHV-5 reference genome with no reads mapping in any sample.

## Discussion

Despite notable advancements in medical technology and techniques, the current state of knowledge regarding inborn errors of immunity remains insufficient. The estimated time from the onset of the first symptoms to the diagnosis of IEI is 16.1 years (2020 CIS annual meeting: immune deficiency & Dysregulation North American conference, 2020). For the most prevalent group of IEIs, namely primary antibody deficiencies, the estimated delay is 6-12 years (Messelink et al., 2023). In the study conducted by the author of this paper, the mean interval between the onset of symptoms and the establishment of an IEI diagnosis was 12.69 ± 13.94 years, with nearly 70% of IEIs diagnosed within the last decade (Napiórkowska-Baran et al., 2023). In the case of the described patient, the delay reached 31 years, as symptoms indicative of IEI first appeared at age 24 as an episode of thrombocytopenia. The age of initial symptom onset is characteristic of CVID, which typically manifests in childhood or precisely in early adolescence (between the ages of 20 and 45) (Carrabba et al., 2023).

Gastrointestinal disorders are relatively common in patients with CVID. In a study conducted by Pikkarainen S. et al., diarrhea and/or weight loss were the most frequent reasons for referring patients to a gastroenterologist (Pikkarainen et al., 2019). The most common pathogens isolated in patients with CVID and diarrhea are *Campylobacter*, *Clostridioides difficile* (toxin), *Giardia* spp., *Salmonella* spp., *Shigella* spp., *Cryptosporidium* spp., *Microsporidium* spp., HHV-5 (*Cytomegalovirus*, CMV), *Norovirus*, *Enterovirus*, and various intestinal parasites (Bethune et al., 2019).

Patients with IEI exhibit an aberrant microbiome, not only in the intestines (Shulzhenko et al., 2018; Fiedorová et al., 2019; Varricchi et al., 2021). In a study conducted by Baniadam L. et al., more than 60% of patients diagnosed with CVID exhibited indications of small intestinal bacterial overgrowth (Baniadam et al., 2021). Some evidence indicates a discrepancy in the composition of the intestinal microflora between CVID patients and healthy individuals. In their study, Bosák J et al. demonstrated that patients with CVID exhibited greater gut bacterial diversity and levels of low-abundance genes than their healthy household members. Significant differences were observed in the relative abundance of thirty-four specific bacterial species. Metagenomic analysis revealed that individuals with CVID differed from their roommates in the abundance of eleven species belonging to the *Firmicutes* and one species among the *Actinobacteria*. Interestingly, they exhibited an increased abundance of species such as *Hungatella*, *Erysipelatoclostridium*, *Tyzzerella*, *Anaerotignum*, and *Anaeromassilibacillus*. At the same time, *Mitsuokella*, *Megasphaera*, *Holdemanella*, *Acidaminococcus*, *Faecalitalea*, *Staphylococcus*, and *Actinomyces* were less abundant in the study group. Additionally, the CVID metagenome exhibited an enrichment of low-abundance genes, which are likely to encode non-essential functions such as bacterial motility and the metabolism of aromatic compounds. Although the study was conducted on a limited number of participants, it demonstrated an expansion of bacterial diversity in the context of host immunodeficiency and identified several bacterial species and metabolites that may serve as diagnostic and/or prognostic markers for CVID in the future (Bosák et al., 2021). Sharma M indicates that patients with CVID have an altered composition of the intestinal microflora, a reduced quantity of beneficial bacteria such as *Bifidobacterium* and *Lactobacillus*, *Bacteroides* and *Firmicutes*, and an increased abundance of *Clostridium* spp., *Bacillus* spp., *Prevotella* and *Gammaproteobacteria* (Sharma et al., 2023), which was also confirmed by the study presented by the authors of this paper. Poto R’s paper highlighted the potential for mitigating dysbiosis in CVID patients to affect clinical outcomes and survival, suggesting this represents a novel avenue for evaluation (Poto et al., 2023).

Moreover, the patient had secondary immunodeficiency, which was not merely a consequence of the immunosuppressive treatment administered but also a result of the splenectomy. The administration of total parenteral nutrition ensured the provision of essential nutrients and non-nutritive bioactive compounds, which are crucial for the synthesis of proteins and cells of the immune system. Splenectomy has a therapeutic effect in numerous conditions, including idiopathic thrombocytopenic purpura (ITP), lymphomas, sickle cell anemia, and thalassemia. Nevertheless, this procedure increases the risk of infection and, in some cases, can lead to severe sepsis, clinically defined as overwhelming post-splenectomy infection (OPSI), which has a very high mortality rate. Enveloped bacteria make up the majority of invasive organisms, the most common of which is *Streptococcus pneumoniae* (Tahir et al., 2020).

Fecal microbiota transplantation is by far the most effective option for restoring gastrointestinal homeostasis, compared to other methods such as antibiotic therapy aimed at eliminating pathogenic bacteria or the use of probiotics (Cammarota et al., 2015; Khoruts, 2018; Minkoff et al., 2023). In 2013, the U.S. Food and Drug Administration approved FMT for the treatment of recurrent and refractory *Clostridium difficile* infections (Wang et al., 2019). Studies also confirm the positive effect of FMT in diseases such as IBD, Parkinson’s disease, and metabolic diseases (Alexandrescu et al., 2024; Sadowski et al., 2024; Zeng et al., 2024). However, these studies concern immunocompetent patients. To the best of our knowledge, the available literature has not yet described the use of FMT in a patient with IEI. This is due to the absence of randomized clinical trials, as well as concerns regarding potential adverse effects. As evidenced by the literature, there have been documented cases of infections caused by probiotic strains of *Lacticaseibacillus rhamnosus* (formerly known as *Lactobacillus rhamnosus*) and *Lacticaseibacillus casei* in patients with secondary immunodeficiencies. The most prevalent clinical manifestations were endocarditis and bacteremia, with peritonitis, abscesses, and meningitis occurring less frequently. Moreover, the overall mortality rate was approximately 30% (Cannon et al., 2005; Mikucka et al., 2022).

Considering the exhaustion of therapeutic options, the treatment of diarrhea by transplantation of intestinal microbiota with the certified Mbiotix HBI preparation from the Human Biome Institute was undertaken after obtaining informed consent from the patient and approval from the bioethics committee to conduct a medical experiment. The preparation is a certified product, and the donor is subjected to a comprehensive examination, a medical interview, and a multi-step qualification process. Additionally, the donor is tested at least twice in each donation cycle, with each material undergoing further testing to exclude the presence of pathogenic microorganisms. The safety of the product has been evaluated in numerous patient groups, including those with steroid-resistant or steroid-dependent graft-versus-host disease (GvHD) secondary to hematopoietic stem cell transplantation (HSCT) (Qiao et al., 2023).

Before each FMT, the patient was given laxatives to maximize the chances of a successful transplant and to ensure the patient’s safety during and after the procedure. This is an important preparatory step that serves several key functions. Laxatives help remove any remaining food residues and stool from the intestines. This creates better conditions for the transplanted microbiota to colonize, as it is not disrupted by the presence of other bacteria and waste. They also reduce bacterial competition. Cleansing the intestines decreases the number of pathogenic bacteria and excess microorganisms that could compete with the new, transplanted microbiota. This allows the transplanted gut bacteria to more easily take hold in the intestinal ecosystem and begin to fulfill their functions. This approach increases the effectiveness of the transplant. The transplanted microbiota has a better chance of successfully establishing itself and integrating with the recipient’s intestines. Studies show that cleansed intestines respond better to microbiota therapy, which enhances the overall success of the procedure. In addition, this approach reduces the risk of infection. Reducing the number of pathogenic bacteria and other microorganisms in the intestines before the transplant microbiota reduces the risk of infection or complications related to bacterial imbalance after the transplant. However, intestinal cleansing serves primarily to remove and flush out bacteria closest to the mucosa and make space in this most immunologically active zone of the microbiome and intestine (Fang et al., 2021; Hamamah et al., 2022; Gulati et al., 2023)..

The patient was not given antibiotics before the second FMT. Before the first FMT procedure, antibiotics are used to reduce the number of pathogens as much as possible and to reduce the number of bacteria in the intestines, which is to make it easier for the transplanted microorganisms to colonize the intestines. However, antibiotics are usually not used before the next FMT procedure. There are several reasons why this is the case. After the first FMT, the recipient’s microbiota is already partially replaced by the healthy donor microbiota. The second procedure is intended to strengthen the newly formed microbiota, not to completely change it. Therefore, antibiotics that could destroy both pathogens and beneficial bacteria are not needed. After the first FMT, the transplanted bacteria begin to grow in the patient’s intestines. Administering antibiotics before the next procedure could damage the beneficial bacteria already colonizing the intestines, which could reduce the effectiveness of the treatment. The idea is not to destroy the healthy microbiota that has already been transplanted. Additionally, the second FMT is more supportive than radical - its aim is to improve or supplement the colonization of the intestines by healthy microbiota. It is therefore not necessary to further eliminate bacteria with antibiotics, as is done before the first treatment (Battipaglia et al., 2019; Wilcox et al., 2020; Popa et al., 2021).

Despite CMV presence confirmed by PCR, its DNA was not found in NGS data, even though a dedicated check for its presence was performed. There are several possible explanations for this. Mainly, the applied method of DNA isolation from feces is designed to isolate mostly bacterial DNA. At the same time, it limits the amount of host and free DNA, both being the main potential sources of HHV-5 DNA in the feces. Furthermore it has been shown that PCRs can be more sensitive than whole metagenome sequencing (Borillo et al., 2022).

Furthermore, in the patient, the genes encoding important virulence factors, i.e., resistance to heavy metals, biocides, heat, and acidic conditions, were initially reduced in quantity and eventually completely eliminated in a test performed after 14 days. The number of genes associated with antibiotic resistance increased over time. Notably, however, these genes’ cumulative abundance (RPKM) decreased.

On top of the reconstruction of the patient’s microbiome after FMT, a reduction in inflammatory parameters (IL-6) was achieved, as well as a notable enhancement in the hemolytic activity of the classical CH50 complement pathway. It is hypothesized that the complement system acts as a protective factor for the host against microbial invasion during infection. Nevertheless, recent studies have indicated that deficiencies in complement-related proteins are associated with the development of various diseases, including autoimmune and inflammatory conditions (Ohtani, 2020). IL-6 seems to be a good marker of inflammation, also occurring in the intestine. Studies show that probiotic strains regulate the secretion of inflammatory cytokines, including IL-6 (Mohammed et al., 2024). Moreover, studies conducted in patients with IBD show that this cytokine can be a marker predicting the therapeutic response in this group of patients (Elhag et al., 2022). It seems that the analysis of patients with IEIs with concomitant IBD, especially in relation to FMT and IL-6, CH50 determinations, could be a new valuable direction of research.

The therapy performed on the patient allowed for colonization with microorganisms characteristic of the normal intestinal microbiome. Homeostasis of the digestive tract is essential for the proper functioning of the entire organism. Without a proper intestinal microbiota, this is not possible. The intestinal microbiome, or the group of microorganisms inhabiting our intestines, plays a key role in the health and functioning of the body. Its role is multidimensional and includes aspects such as digestion and metabolism. The intestinal microbiome acts as a protective barrier, competing with pathogens for space and nutrients. It also produces antimicrobial substances that can inhibit the development of harmful microorganisms. It also stimulates the immune system by affecting dendritic cells, epithelial cells, regulatory T cells, effector lymphocytes, NK cells, B lymphocytes and macrophages. Commensal bacteria, through appropriate signals, can strengthen the integrity of the intestinal epithelium, stimulate the rate of enterocyte proliferation or stimulate the production of mucin. They also stimulate the production of secretory immunoglobulins (sIgA class) and Cationic Antimicrobial Peptides (CAMPs). They provide not only nutrients, but also vitamins K, B1, B6, B12 and folic acid. Intestinal bacteria can affect the metabolism of drugs and toxins, which is important for the effectiveness of pharmacotherapy and detoxification of the body. *Enterococcus* spp. have the ability to induce the expression of genes responsible for the production of IL-10, which has anti-inflammatory effects. A healthy microbiome plays an invaluable role (Lloyd-Price et al., 2016; Ahrodia et al., 2022).

## Conclusions

The presented case illustrates the potential of FMT as a novel and safe therapeutic option for patients with IEI. One limitation of the method’s use is the lack of randomized trials and the necessity to obtain approval from the bioethics committee to conduct a medical experiment. The method is safe, although it necessitates more frequent medical interviews, physical examinations (including body temperature observation), and monitoring of inflammatory markers in immunocompromised patients. A growing body of evidence from extensive studies of the microbiome is elucidating the relationship between gastrointestinal homeostasis and certain chronic diseases. It is feasible that, in addition to the treatment of gastrointestinal diseases, this will prove to be an effective option for the prevention and treatment of patients with IEI.

## Data Availability

The raw data supporting the conclusions of this article will be made available by the authors, without undue reservation.
